# Tethering telomerase to telomeres increases genome instability and promotes chronological aging in yeast

**DOI:** 10.18632/aging.101095

**Published:** 2016-11-13

**Authors:** Jun Liu, Ming-Hong He, Jing Peng, Yi-Min Duan, Yi-Si Lu, Zhenfang Wu, Ting Gong, Hong-Tao Li, Jin-Qiu Zhou

**Affiliations:** ^1^ The State Key Laboratory of Molecular Biology, CAS Center for Excellence in Molecular Cell Science, Innovation Center for Cell Signaling Network, Institute of Biochemistry and Cell Biology, Shanghai Institutes for Biological Sciences, Chinese Academy of Sciences, University of Chinese Academy of Sciences, Shanghai 200031, China; ^2^ School of Life Science and Technology, Shanghai Tech University, Shanghai 201210, China

**Keywords:** chronological aging, CDC13-EST2 fusion, genome instability, Sch9, Rif1, yeast

## Abstract

Chronological aging of the yeast *Saccharomyces cerevisiae* is attributed to multi-faceted traits especially those involving genome instability, and has been considered to be an aging model for post-mitotic cells in higher organisms. Telomeres are the physical ends of eukaryotic chromosomes, and are essential for genome integrity and stability. It remains elusive whether dysregulated telomerase activity affects chronological aging. We employed the *CDC13-EST2* fusion gene, which tethers telomerase to telomeres, to examine the effect of constitutively active telomerase on chronological lifespan (CLS). The expression of Cdc13-Est2 fusion protein resulted in overlong telomeres (2 to 4 folds longer than normal telomeres), and long telomeres were stably maintained during long-term chronological aging. Accordingly, genome instability, manifested by accumulation of extra-chromosomal rDNA circle species, age-dependent *CAN1* marker-gene mutation frequency and gross chromosomal rearrangement frequency, was significantly elevated. Importantly, inactivation of Sch9, a downstream kinase of the target of rapamycin complex 1 (TORC1), suppressed both the genome instability and accelerated chronological aging mediated by *CDC13-EST2* expression. Interestingly, loss of the *CDC13-EST2* fusion gene in the cells with overlong telomeres restored the regular CLS. Altogether, these data suggest that constitutively active telomerase is detrimental to the maintenance of genome stability, and promotes chronological aging in yeast.

## INTRODUCTION

Aging is broadly defined as a time-dependent functional decline that most living organisms seem to be unable to escape. It is one of the leading risk factors for a number of aging-associated diseases including atherosclerosis, type 2 diabetes, cardiovascular diseases, cancer and Alzheimer's disease [1]. Several lines of evidence suggest that slowing down aging process reduces the incidence of age-related diseases and extends organismal lifespan and health span [2, 3]. Most of the mechanistic studies on aging have involved various model organisms, including multicellular worm, fly, mouse, monkey, as well as single-cellular yeast (e.g. *Saccharomyces cerevisiae*). Two forms of lifespan in yeast *S. cerevisiae* are usually used to describe aging, namely replicative lifespan (RLS) and chronological lifespan (CLS) [4]. The RLS measures the exact number of daughter cells produced by a single mother cell before cell death [5]. CLS is defined as the length of time that the cells at stationary phase or G_0_ phase (quiescence state) can be viable and reenter cell cycle upon nutrient availability [6]. The replicative aging models the aging process of dividing cells, while the chronological aging mirrors the aging process of non-dividing (post-mitotic) cells. Numerous studies on yeast chronological aging have been accelerating the pace of revealing the mystery of aging and longevity [4, 7–13]. Two evolutionarily conserved nutrient-sensing signaling pathways, the Tor (target of rapamycin)/Sch9 pathway and Ras/PKA pathway, are first demonstrated to modulate CLS in the budding yeast *S. cerevisiae* [14]. These two signaling pathways are later found to regulate lifespan in higher organisms including mammals as well [2, 15–18]. Sch9 is a direct downstream kinase of TORC1 (the target of rapamycin complex 1) [19]. Deletion of *SCH9* in yeast inhibits genome instability during chronological aging, and results in significant increase of CLS [14]. The evolutionarily conserved Tor/Sch9 pathway and Ras/PKA pathway also regulate replicative aging [4, 20].

Genome instability, one of the hallmarks of aging [1, 21], is deleterious for maintaining a long lifespan in many organisms ranging from yeast to mammals [8, 21–25]. In *S. cerevisiae*, the rDNA stability is suggested to regulate both replicative and chronological aging [26–28]. Sir2 and Fob1 play opposite role in rDNA recombination, i.e. Sir2 inhibits, while Fob1 promotes rDNA recombination [29]. Multimer ERC accumulation is significantly enhanced in mutants that have aberrant cell cycle checkpoint control during chronological aging [26]. Sgs1 is a DNA helicase, whose deletion causes much higher gene mutation frequency and gross chromosomal rearrangement (GCR) frequency than wild-type cells during chronological aging [22], but does not result in elevated level of ERCs [30–32]. The phenotypes of genome instability can be suppressed by *SCH9* deletion [22]. The Bloom syndrome helicase (BLM) and Werner's syndrome helicase (WRN) are mammalian orthologs of yeast Sgs1. They are involved in the maintenance of genome stability [22, 33]. Mutations in human BLM or WRN may cause pre-mature aging syndrome [34].

Telomeres, the protective DNA-protein structures at the ends of eukaryotic chromosomes, are essential for genome integrity and stability [35]. In *S. cerevisiae*, telomeric DNA consists of ~350 bp of TG_1–3_/ C_1–3_A repeats. The G strand extends beyond its complementary strand to form a single-stranded over-hang (called G-overhang) [36]. Telomeric DNA is mainly elongated by a specialized reverse transcriptase called telomerase. Telomerase consists of at least four subunits, the catalytic protein subunit Est2, the RNA template subunit Tlc1 and two accessory subunits Est1 and Est3. Cdc13 is a telomeric single stranded DNA binding protein, which associates with telomeres throughout the cell cycle [37]. While Est1 abundance is cell cycle regulated, which binds telomeres late in S phase. Est1 may interacts with Cdc13 in S phase to convert inactive telomerase to active form [37–40]. The expression of fusion protein Cdc13-Est2 forces telomerase to be constitutively tethered to telomeres, and leads to progressive and over elongation of telomeres [41]. Rif1 and Rif2 are two negative regulators of telomerase [42, 43]. Deletion of either *RIF1* or *RIF2* causes telomere lengthening but *rif1*Δ cells have much longer telomeres than *rif2*Δ cells [42, 43].

In yeast the absence of telomerase leads to shortening of telomeric TG_1-3_ DNA at a rate of 2.5-5 base pairs per population doubling [44], and telomerase-null cells eventually cease to divide when telomeres reach critically short length, resulting in replicative senescence [45], which represents a different aging model from the telomere-length indenpendent replicative aging and chronological aging aforementioned. In most mammalian somatic cells, low or no telomerase activity also results in gradual attrition of telomeres and cellular or organismal aging [46–48]. Conversely, experimental telomere lengthening is correlated with increased lifespan in mice [49, 50]. Polymorphisms in telomere maintenance factors that lead to longer telomeres are associated with diminished age-related pathology in humans [51]. Additionally, transient overexpression of hTERT in human cells lengthens telomeres and extends RLS [52]. Moreover, overexpression of a telomere binding protein HRP-1 in the nematode *Caenorhabditis elegans* lengthens lifespan [53]. These findings intuitively suggest that long telomeres might be beneficial to lifespan extension. However, the effect of telomere length on the lifespan regulation remains controversial. Telomere length is stably maintained during replicative aging of yeast cells [54]. The yeast cells that had overlong telomeres displayed a regular RLS [23], while a truncated *tlc1* mutant strain that had shorter but stable telomeres exhibited longer RLS than the wild-type strain [55]. Interestingly, the increase of telomere length in mouse tissues of spleen, colon and liver appears to be associated with chronic inflammation accelerated aging [56]. Long telomeres also associated with increased risk for pulmonary hypertension [57]. Nevertheless, how dysregulated telomerase activity influences yeast CLS is still unknown. In this work, we have employed the fusion protein Cdc13-Est2 to force constitutive association of telomerase with telomeres, and explore the effect of constitutive telomerase activity on CLS. Our results suggest that constitutively active telomerase results in genome instability, which accelerates yeast chronological aging.

## RESULTS

### Genetic strategy to obtain the cells with overlong telomeres

**Figure 1:**
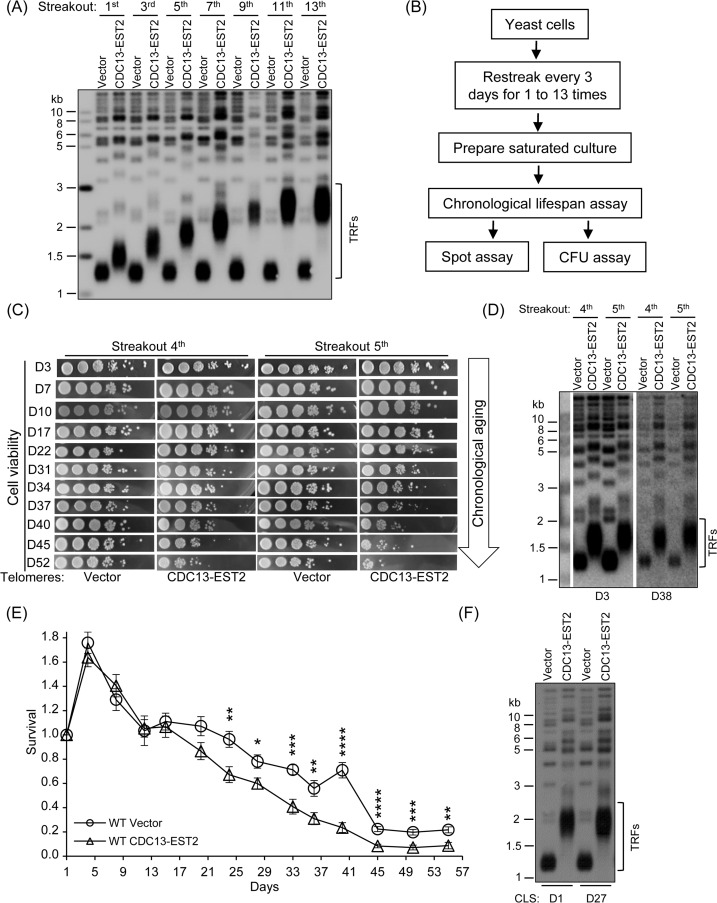
Expression of *CDC13-EST2* promotes yeast chronological aging (**A**) Telomere length analysis by Southern blot. The cells that contain *pRS316* (Vector) or *pRS316*-*CDC13-EST2* plasmid were passaged, and the genomic DNA from cells of different streakouts (labeled on top) was subjected to Southern blot assay using a telomeric TG_1-3_ probe. (**B**) Flow chart of CLS assay. (**C**) Spot assay of CLS. Both the normal-telomere and overlong-telomere cells of streakouts 4^th^ and 5^th^ were used to perform semi-quantitative CLS assay. The time of cultures in the CLS assay was labeled on the left (e.g. D3 means day 3). (**D**) Telomere length of cells used in (**C**) at D3 and D38 was examined by Southern blot with telomeric TG1-3 probe. (**E**) Colony formation unit (CFU) assay of CLS. CLS of cells BY4742-pRS316 (streakout 8^th^, normal telomeres) and BY4742 -pRS316-CDC13-EST2 (streakout 8^th^, overlong telomeres) was quantitatively examined. Survival (viable colonies) values at the indicated days of culture were normalized to CLS D1. Values are the averages of 6-10 cultures ± SEM. * denotes p<0.05, ** p<0.01, *** p<0.001 and **** p<0.0001. (**F**) Telomere length of cells in (**E**) at CLS D1 and CLS D27 was examined by Southern blot.

In the budding yeast *S. cerevisiae*, telomeres are usually 350 ± 75 bp long. The telomere length is mainly determined by two opposite activities: telomerase-mediated elongation and nuclease-mediated degradation. In order to obtain the strains that have overlong telomeres, we employed the *CDC13*-*EST2* fusion gene, whose expression can force enhanced association of telomerase at telomeres and produce long telomeres after successive passages [41]. We transformed either the *CEN* plasmid *pRS316* (control) or the plasmid containing the *CDC13*-*EST2* fusion gene, namely *pRS316-CDC13-EST2*, into the wild-type BY4742 cells. The individual transformants were passaged on selective plates every 72 hrs for thirteen times. The telomere length was examined by Southern blot using a telomeric TG-probe. The result showed that the ectopic expression of Cdc13-Est2 fusion protein led to gradual elongation of all telomeres (Fig. 1A). At the 5^th^ and 13^th^ streakout of transformants with *CDC13-EST2* fusion gene, the size of the telomeric terminal-restriction-fragments (TRFs) digested by restriction endonuclease *Xho*I reached about 2.0 and 2.6 kb, respectively, which was 0.7 and 1.3 kb longer than that of the vector control, and 0.5 and 1.1 kb longer than that of the 1^st^ streakout. The telomere TRFs longer than 2.0 kb were designated “overlong telomeres”. Because the TRFs contain about 0.95 kb subtelomeric sequence, and the telomeric TG_1-3_-tracts of wild-type cells are about 0.35 kb, the TG_1-3_-tracts of the Cdc13-Est2 expressing cells are approximately two (the 5^th^ streakout) to four (the 13^th^ streakout) folds longer.

### Expression of Cdc13-Est2 promotes yeast chronological aging

To evaluate the effect of Cdc13-Est2 expression on CLS, we randomly chose multiple single colonies to perform CLS assay. The procedures for a CLS experiment were schematically shown in Fig. 1B (see Materials and Methods for the details) [58, 59]. The results showed that along the extension of culture time (day 3 to day 52), the viability of the cells with Cdc13-Est2 expression dropped much faster than that of the cells without Cdc13-Est2 expression (Fig. 1C). Because the CLS assay involved many steps, as well as many days of culturing (Fig. 1B and C), there was a possibility that telomere length had changed in the long process of culture. Therefore, we examined the telomere length in the cells of day 3 and day 38 samples by Southern blot (D3 and D38 in Fig. 1D). The results showed that the telomeres in the cells of day 38 had not undergone lengthening or shortening (Fig. 1D), and the telomeres in the Cdc13-Est2-expressing cells were substantially longer than that in the cells containing the control-vector. These data suggested that overlong telomeres induced by Cdc13-Est2 expression led to shorter CLS.

To quantify the survival rate of the aging cells, we carried out the colony formation assay (see the Materials and methods for the details) [7, 59]. Multiple single colonies of the 8^th^ streakout were used to perform the CLS assay. The survival rate of the cells expressing *CDC13-EST2* fusion gene was significantly lower than that of the cells containing the control vector (Fig. 1E). The telomere length in the tested cells did not change during the long-time culture (e.g. 27 days, D27 in Fig. 1F). These results consistently supported the notion that Cdc13-Est2 expression results in overlong telomeres and promotes yeast chronological aging.

### Expression of Cdc13-Est2 enhances ERC accumulation

**Figure 2:**
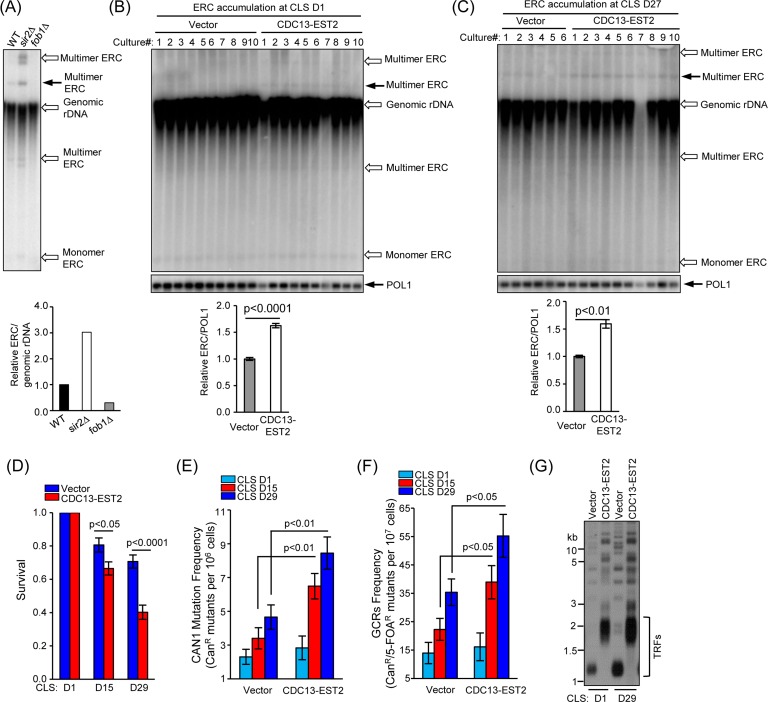
*CDC13-EST2* expression enhances genome instability (**A**) ERC level in *fob1*Δ and *sir2*∆ mutant cells was examined by Southern blot with a probe of 25S rDNA sequence (upper panel). One species of multimer ERCs (indicated by solid arrow) was quantified and normalized to genomic rDNA signal. Lower panel is the quantification of the ERC level in *fob1*∆ and *sir2*∆ mutant cells. The ERC level in wild-type cells was set as “1”, and the ERC value in each strain was normalized to the genomic rDNA. (**B**) and (**C**) Detection of ERC level in young cells of CLS D1 (B) and old cells of CLS D27 (C) used in Fig. 1E by Southern blot (upper panel). One species of multimer ERC (indicated by solid arrow) was quantified (lower panel). *POL1* gene was used as an internal loading control. Values in the quantification are normalized to *POL1* level ± SEM. (**D**) Chronological viability of cells carrying *CAN1*-*URA3* cassette at CLS D1, D15 and D29. The cells of streakout 8^th^ containing pRS315 (control) and pRS315-*CDC13-EST2* was examined. Survival (viable colonies) values were normalized to CLS D1. Values are the averages of 6-10 cultures ± SEM. (**E**) and (**F**) *CAN1* mutation frequency (**E**) and GCRs frequency (**F**) in overlong- and normal-telomere cells at CLS D1, D15 and D29 were examined. Values in (**E**) and (**F**) are the averages of 6-10 cultures ±SEM. (**G**) Telomere length analysis of cells at CLS D1 and D29. The cells of streakout 8^th^ that containing pRS315 (normal telomere) and pRS315-*CDC13-EST2* (overlong telomere) were examined.

Genome instability is one of the major regulators of CLS. Constitutively active telomerase at telomeres might have led to genome instability that shortens CLS. To test this hypothesis, we firstly examined the recombination activity of rDNA loci because the production of extra-chromosomal rDNA circles (ERCs) is an indication of rDNA stability [27]. The *fob1*Δ and *sir2*Δ cells were used as the negative and positive controls, respectively (see Materials and Methods for detail). The Southern blot results showed that deletion of *FOB1* and *SIR2* reduced and increased the ERCs, respectively (Fig. 2A). Six to ten independent cultures of both the young (CLS D1) and old (CLS D27) cells were subjected to ERC detection (Fig. 2B and C). The ERCs level in the *CDC13-EST2* expressing cells is significantly higher than that in the vector-control cells (Fig. 2B and C). One species of multimer ERCs (indicated by solid arrow) in either the young (CLS D1) or old *CDC13-EST2* expressing cells (CLS D27) was approximately 60% higher than that in the vector-control cells (Fig. 2B and C). The enhanced ERCs levels in young (CLS D1) and old (CLS D27) cells expressing *CDC13-EST2* were comparable, we speculated that ERCs accumulation might take place in the proliferation stage of the culture (Fig. 2B and C). These results suggested that Cdc13-Est2 expression perturbed genome stability.

### Expression of Cdc13-Est2 increases age-dependent *CAN1* marker-gene mutation frequency and GCR frequency

In order to validate further our hypothesis that expression of Cdc13-Est2 promotes yeast chronological aging by increasing genome instability, we next examined the *CAN1* marker-gene mutation frequency and gross chromosomal rearrangement (GCR) frequency in the *CDC13-EST2* expressing cells. These two assays have been widely used to evaluate age-dependent genome instability in yeast [8, 22, 60, 61].

The *CAN1* gene encodes an arginine permease, which controls the uptake of arginine by yeast cells. Detection of the *CAN1* mutation frequency uses an arginine analogue, canavanine, which cells intake under arginine deficient conditions. Canavanine is toxic to yeast cells and leads to cell death. Unless *CAN1* mutation (such as point mutation, insertion or deletion) inactivates *CAN1*, cells cannot survive when canavanine is supplemented in the medium lack of arginine [60]. Different from the *CAN1* marker-gene mutation frequency assay, the GCR assay detects the loss of both *CAN1* and *URA3* marker genes simultaneously [60]. To measure GCR, the *CAN1* proximal to *HXT13* gene was substituted by *URA3* marker-gene. 5-fluoroorotic acid (5-FOA) was used to counter-select yeast cells expressing *URA3*. In the presence of both 5-FOA and canavanine, cells must lose the functions of both *CAN1* and *URA3* to survive. We firstly examined the survival rate of the cells that contained the *CAN1-URA3* marker cassette. The results showed that *CDC13-EST2* expressing cells had significantly shortened CLS compared with vector control cells at CLS D15 (p value <0.05, n=6-10) and CLS D29 (p value <0.0001, n=6-10) (Fig. 2D). Consistently, *CDC13-EST2* expression significantly increased the *CAN1* marker-gene mutation frequency at CLS D15 (p value <0.01, n=6-10) and CLS D29 (p value <0.01, n=6-10) (Fig. 2E), and the GCR frequency at CLS D15 (p value <0.05, n=6-10) and CLS D29 (p value <0.05, n=6-10) (Fig. 2F). The cells used to detect *CAN1* mutation frequency and GCR frequency did have overlong telomeres, and their long telomeres were stably maintained during chronological aging (Fig. 2G). Altogether, our results indicated that Cdc13-Est2 expression caused genome instability, and accelerated chronological aging.

### Deletion of *SCH9* reduces genome instability and suppresses chronological aging mediated by expression of Cdc13-Est2

Previous study has shown that deletion of *SCH9* in yeast significantly decreases genome instability during chronological aging [22]. Now that Cdc13-Est2 expression led to enhanced genome instability and resulted in accelerated chronological aging, could calorie restriction (CR) or deletion of *SCH9* slow down the rate of chronological aging mediated by Cdc13-Est2 expression? To address this question, we examined whether reducing glucose concentration from 2% to 0.5%, which mimics moderate CR conditions, could restore the CLS of the *CDC13-EST2* expressing cells. The results showed that moderate CR did not significantly affect telomere length (Fig. S1A), nor recover the CLS of *CDC13-EST2* expressing cells (Fig. S1B and C). This result is consistent with previous report that buffering culture medium to pH 6.0 by citrate/phosphate acts similarly with moderate CR in lengthening CLS [62], and application of moderate CR to synthetic complete medium buffered to pH 6.0 with citrate/phosphate cannot further extend CLS [62].

We next examined the effect of deletion of *SCH9* on chronological aging in *CDC13-EST2* expressing cells.

**Figure 3:**
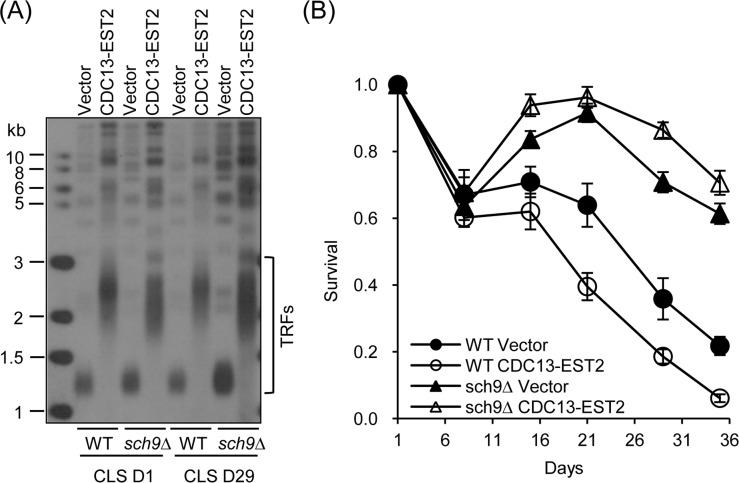
CLS of sch9∆ cells (**A**) Telomere length of cells in (**B**) at CLS D1 and CLS D29 was examined by Southern blot. (B) CLS assay was done using cells WT-pRS316/sch9∆-pRS316 (streakout 13^th^, normal telomeres), WT-pRS316-CDC13-EST2/sch9∆-pRS316-CDC13-EST2 (streakout 13^th^, overlong telomeres). Survival (viable colonies) values are normalized to CLS D1 and are the averages of 6-10 cultures ± SEM.

**Figure 4:**
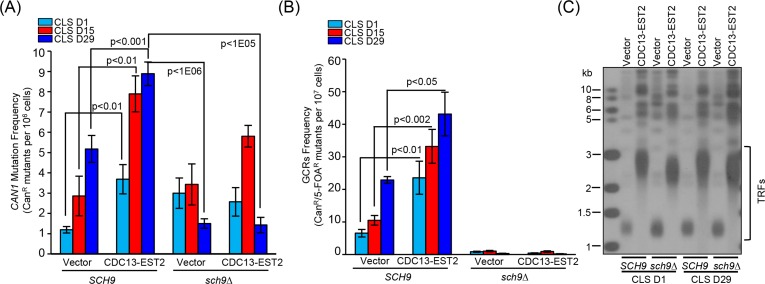
Detection of *CAN1* mutation frequency and GCR frequency in *sch9*∆ cells during chronological aging (**A**) Cells of *hxt13*∆-pRS315/ *hxt13*∆*sch9*∆-pRS315 (streakout 14^th^, normal telomeres), *hxt13*∆-pRS315-CDC13-EST2/ *hxt13*∆*sch9*∆-pRS315-CDC13-EST2 (streakout 14^th^, overlong telomeres) were used to assay *CAN1* marker-gene mutation frequency during chronological aging. (**B**) The same cells in (**A**) were used to examine GCRs frequency during chronological aging. The values (viable colonies) are the averages of 6-10 cultures ± SEM. (**C**) Telomere length of cells of CLS D1 and D29 in (**A**) and (**B**) was examined by Southern blot.

Deletion of *SCH9* had little effect on telomere length in either vector-control or *CDC13-EST2* expressing cells (Fig. 3A), but significantly extended CLS in both cells (Fig. 3B). This is consistent with published data that *sch9*∆ increases CLS [14]. In order to address whether the CLS recovery is attributed to the suppression of genome instability, we examined *CAN1* marker-gene mutation frequency, GCR frequency and ERCs accumulation in the *sch9*Δ cells with *CDC13-EST2* expression. The results showed that deletion of *SCH9* significantly lowered both the *CAN1* marker-gene mutation frequency and the age-dependent GCR frequency mediated by *CDC13-EST2* expression during chronological aging (Fig. 4A and B). The cells in the genome instability assay did have overlong telomeres and the telomere length did not exhibit a significant change during chorological aging (e.g. CLS D29) (Fig. 4C). In addition, deletion of *SCH9* also lowered ERC accumulation mediated by *CDC13-EST2* expression at CLS D1 (p value < 9.2E-07, n=6) and CLS D15 (p value < 8E-05, n=6) (Fig. S2A and B). *CDC13-EST2* expressing cells stably maintained overlong telomeres during chronological aging (Fig. S2C). These data suggested that deletion of *SCH9* contributed to the maintenance of genome stability in Cdc13-Est2 expressing cells, and inhibition of genome instability by deletion of *SCH9* slowed down chronological aging process promoted by constitutively active telomerase.

### Loss of *CDC13*-*EST2* fusion gene restores regular CLS

**Figure 5:**
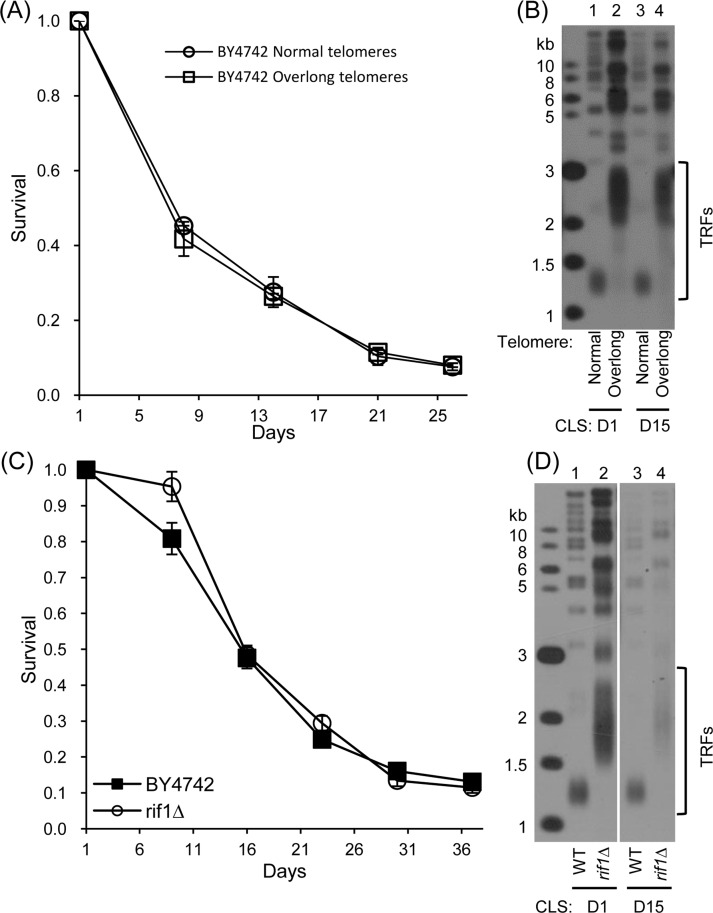
CLS assay of cells with overlong telomeres but no *CDC13-EST2* fusion gene (**A**) CLS of normal- and overlong-telomere cells after plasmids eviction (21^st^ streakout) (**B**) Southern blot analysis of telomere length using telomeric TG_1-3_ probe. (**C**) CLS of wild-type (4^th^ streakout) and *rif1*∆ (4^th^ streakout). (**D**) Telomere length detection by Southern blot at CLS D1 and D15.

We used a genetic strategy to force enhanced association of telomerase at telomeres through expression of *CDC13*-*EST2*. The expression of Cdc13-Est2 fusion protein resulted in overlong telomeres and shortened CLS (Fig. 1). However, it was unclear whether the shortened CLS was attributed to overlong telomeres or constitutive association of telomerase at telomeres. To discriminate these two possibilities, the pRS316 (control) and pRS316-*CDC13*-*EST2* plasmids in the normal- and overlong-telomere cells were evicted, and CLS assays were performed (See Fig. S3 and S4 for the details). The results showed that the CLS of cells with overlong telomeres was not different from that of cells with normal telomeres (Fig. 5A and S4C). The cells used in the CLS assays maintained either normal or overlong telomeres during their long-term chronological aging (Fig. 5B and S4D). These results suggested that it was not the overlong telomeres, but rather the constitutive association of telomerase at telomeres that affected CLS. Following the notion that overlong telomeres might not affect CLS, we examined CLS of the *rif1*∆ mutant cells that have overlong telomeres (Fig.5D) [42, 43]. The results showed that the *rif1*∆ mutant cells exhibited nearly the same chronological aging profile as the wild-type cells (Fig.5C). The telomere length of wild-type and *rif1*∆ cells was stably maintained during chronological aging (Fig. 5D). Thus, constitutively tethering telomerase to telomeres causes genome instability and promotes chronological aging.

## DISCUSSION

**Figure 6:**
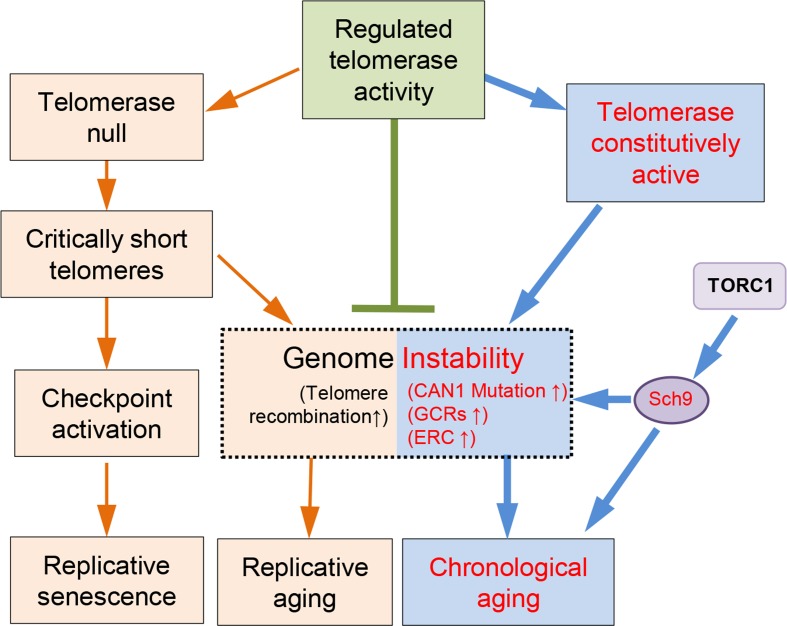
A model of telomere/telomerase associated genome instability affects yeast aging Wild-type yeast cells have evolved to have regulated telomerase activity to maintain telomere homeostasis. In the absence of telomerase, telomeres gradually shrink to critically short ones that lead to either checkpoint activation and replicative senescence, or telomere recombination and accelerated replicative aging. When telomerase is constitutively active, the genome becomes less stable, and promotes chronological aging, which can be suppressed by down-regulation of TORC1/Sch9 pathway.

Previous studies have indicated that the replicative aging and the chronological aging are quite different [4, 63]. However, numerous lines of evidence have pointed to a consensus that genome instability is one of the major culprits of aging (Fig.6) [1]. Telomeres reside at the end of the chromosomes, and their structural integrity is essential for genome stability [37, 64].

Telomerase mediated telomere elongation usually takes place in the late S-phase of the cell cycle, and plays important roles in both telomere length and structure regulation [65, 66]. Intact telomeres are required for genome stability (Fig.6). In the absence of telomerase, following every cell division telomere attrition occurs because of the end replication problem [44, 48, 67]. When telomeres become critically short, cells could either undergo senescence [45], or repair telomeres through homologous recombination that promote replicative aging (Fig.6) [23]. Consistently, long telomeres potentiate longer RLS in human somatic cells that have inactive telomerase [52, 68]. Therefore, longer telomeres appear to be beneficial to lifespan extension when telomerase is not active. In the presence of telomerase, the telomere length and the RLS seems not to have consistent positive correlation. For examples, the mutant cells containing a truncated *tlc1* allele have shortened telomeres but increased RLS, whereas *rif1*∆ mutant cells with long telomeres had shortened RLS [55]. Additionally, overlong telomeres induced by expressing *CDC13-EST2* fusion gene do not affect RLS [23]. In the current work, we have evaluated the effect of overlong telomeres on yeast CLS. Our data suggest that overlong telomeres *per se* do not affect chronological aging (Fig. 5).

Interestingly, constitutively active telomerase mediated by expression of Cdc13-Est2 promotes chronological aging (Fig.1). The underlying reason appears to be the enhanced genome instability (Fig.6), exemplified by increased accumulation of ERCs (Fig. 2A-C), enhanced age-dependent spontaneous *CAN1* marker-gene mutation frequency and GCR frequency in *CDC13-EST2* expressing cells (Fig. 2D-G). How the enhanced association of telomerase at telomeres causes the effect on other genomic loci remains unknown. One possibility is that Cdc13-Est2 fusion protein not only targets to telomeric G-rich overlong, but also spontaneously binds other genomic loci where double strand breaks occur. This speculation is supported by the previous observation that Cdc13 can act on double strand breaks (DSBs) at low frequencies [69, 70]. Under normal situation, Est2 binding to DSBs requires the interaction between Cdc13 and Est1 [71]. Cells seem to have evolved mechanisms that prevent the pervasive action of telomerase on DSBs. For example, Mec1 inhibits telomere healing by phosphorylating Cdc13 on its S306 residue, which suppresses Cdc13 accumulation at DSBs [72]. Est1 abundance is cell cycle regulated and peaks in S-phase, which may limit the activation of Cdc13-bound telomerase Est2 [65]. While under the scenario of Cdc13-Est2 fusion, wherever Cdc13 binds, Est2 may pose an effect on these loci. Telomerase mediated chromosomal healing on one hand repairs the DSBs, but on the other hand may endanger genome stability by loss of chromosomal arms.

The genome alterations observed in the *CDC13-EST2* expressing cells are the accumulation of ERCs (Fig. 2B and C), enhanced spontaneous *CAN1* mutation frequency and GCR frequency (Fig. 2E and F). These changes may exacerbate the chronological aging when the cells are challenged by nutrient depletion (Fig. 1), supporting the argument that genome instability is a direct causal factor for the accelerated chronological aging [1, 22]. In agreement with this hypothesis, deletion of *SCH9* did not shorten the overlong telomeres (Fig. 3A, 4C and S2C), but significantly suppressed the phenotypes of genome instability (Fig. 4A-B and S2A-B), and accordingly restored the lifespan of *CDC13-ETS2* expressing cells (Fig. 3B). The accelerated chronological aging seen in *CDC13-EST2* expressing cells is very similar to the case of *sgs1*Δ mutant cells. Deletion of *SGS1* results in premature age-related changes including reduced CLS, elevated recombinant errors and age-dependent increase in DNA mutations [22]. Additionally, deletion of *SCH9* in *sgs1*∆ mutant cells suppresses recombination and DNA damage, and slows down the chronological aging [22]. Thus, it is significant for the cells to regulate telomerase activity in a properly spatio-temporal manner to avoid genome instability and promote cell longevity.

## MATERIALS AND METHODS

### Yeast strains and plasmids

All the yeast strains used in this study were derived from BY4742 (Euroscarf). The isogenic gene knockout mutant was constructed by one-step gene mutagenesis (Table S1) (See Supplemental information for the details).

### Genomic DNA isolation and Southern blot analysis of yeast telomere length

Genomic DNA isolation and Southern blot analysis were done as what we did previously with minor modifications [73].

### Chronological lifespan assay

Chronological lifespan assay was carried out as previously reported [7, 59, 62]. Glycerol stocks of yeast strains at −80°C freezer were streaked onto fresh YPD or appropriate selective plate and grown for 2-3 days at 30°C. To measure chronological viability, 5-6 singe colonies were suspended into 5 ml of SDC-(amino acids auxotroph requirements) citrate/phosphate buffered medium with pH 6.0 for overnight. A calculated quantity of cells was diluted into 25 ml of SDC medium in a 100 ml flask to give an initial concentration of A600_nm_≈0.005. After 5-7 days of incubation at 30°C with constant shaking at 220 rpm, cells entered into stationary (G_0_) phase. 50 μl of chronologically aged culture was diluted for 2.5×10^5^ folds for CLS D1. 100 μl of dilution was plated onto fresh YPD plate. Cell colony number was counted after 2-3 days of growth at 30°C.

### ERC level determination

ERC detection during chronological aging was performed as previously described [27]. The probe of 25S rDNA sequence (RDN25) (paired primers RDN25-Fwd and RDN25-Rev) for Southern blot detection of ERC was PCR amplified, and was radio-labeled with ^32^P-dCTP. The *POL1* (YNL102W) was used as an internal loading control [23]. The DNA was separated in a 0.7% agarose gel under constant voltage of 60V (2V/cm) for 24 hours and transferred onto a positive charged nylon membrane (GE Healthcare, Cat#: RPN303B).

### *CAN1* marker-gene mutation frequency and GCR frequency detection

*CAN1* marker-gene mutation and GCR frequency were assayed as previously reported with slight modifications [8, 22, 60]. (See Supplemental information for the details).

### Statistical significance calculation

Statistical significance in this study was calculated by the two-tailed student's t test in an Excel spreadsheet.

## SUPPLEMENTARY MATERIAL FIGURES AND TABLES


