# Supplementary material for: Tethering telomerase to telomeres increases genome instability and promotes chronological aging in yeast
Source: Aging (Albany NY). 2016 Nov 13;8(11):2827–40. doi: 10.18632/aging.101095 (PMC5191873; doi:10.18632/aging.101095)
Supplement: Supplementary file 1 [file aging-08-2827-s001.pdf]

SUPPLEMENTARY MATERIAL

Yeast strains and plasmids

The sch9Δ mutant was constructed by transformation of a sch9Δ::HIS3MX6 cassette into BY4742 cells expressing plasmid pRS316-CDC13-EST2, which was amplified by PCR using pFA6a-HIS3MX6 as template, forward primer sch9-UP45-HIS3MX6-F1 and reverse primer sch9-DW45-HIS3MX6-R1. The resulted sch9Δ mutant was confirmed by PCR using three pairs of primers SCH9-5′ UTR-Fwd & HIS3MX6-Rev, SCH9-3′ UTR-Rev & HIS3MX6-Fwd and SCH9-ORF-Fwd & SCH9-ORF-Rev. The mutant hxt13Δ::URA3 (Chen et al., 2009) was validated by PCR using two pairs of primers HXT13-5UTR & URA3-ORF-Fwd and HXT13-3UTR-Rev & URA3-ORF-Rev. The mutant *fob1*Δ::KanMX4 and *sir2*Δ::KanMX4 are further confirmed by paired primers to amplify gene ORF region and gene-specific UTR and KanMX4 region. The *rif1*Δ mutant cells (Euroscarf) were PCR confirmed with two pairs of primers including RIF1-ORF-Fwd & RIF1-ORF-Rev and RIF1-5UTR & KanMX4-ORF-Fwd. To construct pRS315-CDC13-EST2 fusion plasmid, fragment CDC13-EST2 (*Sal*I and *Not*I) from double digestion of pRS316-CDC13-EST2 by *Sal*I and *Not*I was subcloned into pRS315 (*Sal*I and *Not*I) using a ligation high kit (TOYOBO).

CAN1 marker-gene mutation frequency and GCR frequency detection

CAN1 marker-gene mutation and GCR frequency were assayed as previously reported with slight modifications (Huang et al., 2012; Madia et al., 2008; Wei et al., 2011). A non-essential gene *HXT13* proximal to *CAN1* was replaced by marker-gene *URA3*, which can be counter-selected by using 5-FOA. At different time-points of a CLS assay, cell viability was determined by CFU method followed by plating appropriate quantity of cells onto plates without arginine but supplemented with L-canavanine (Sigma Cat#: C9758) (60 mg/L) (for *CAN1* marker-gene mutation frequency), or plates without arginine but supplemented with both L-canavanine (60 mg/L) and 5-FOA (Toronto research chemicals, Cat#: F595000) (1g/L) (for GCR frequency). Viable colony number was counted after 4 days of incubation at 30°C. *CAN1* mutation frequency was calculated as the ratio of colony number on plate with canavanine per ml to the number of cells viable per ml. GCR frequency was determined by colony number on plate supplemented with both canavanine and 5-FOA per ml divided by the number of viable cells per ml.

Genomic DNA isolation and Southern blot analysis of yeast telomeres

About 100 µl cell pellet at different time-points in a chronological lifespan assay were lysed with 500 µl lysis buffer (1% SDS, 100 mM NaCl, 10 mM Tris-HCl pH 8.0, 1mM EDTA) and 100 µl glass beads rigorously in a 1.5 ml centrifuge tube at the maximal speed for 10 min at 4 °C cold room. After 5 min of centrifuge at 13000 rpm at 4°C, supernatant was transferred to a new 1.5 ml centrifuge tube and mixed well with 250 µl 7.5 M ammonium acetate (pH 7.0) followed by 15 min incubation in a 65 °C water bath. Put on ice for another 15 min. Add 500 µl mixture of phenol-chloroform-isoamylic alcohol (25:24:1) (GENERAY BIOTECH, Cat#:GR2516) and mix well by inverting tubes gently and centrifuge at 13000 rpm for 5min. Supernatant was transferred to a new 1.5 centrifuge tube and 500 µl trichloroethane ($CHCl_3$) was added and followed by centrifugation at 13000 rpm for 15 min at 4°C. Transfer again supernatant to a new 1.5 ml centrifuge tube, and add 2-fold volume of absolute alcohol and incubate at -80°C for at least 20 min. Centrifuge at 13000 rpm for 5min at 4 °C, decant supernatant and wash once with 70% ethanol. Finally, air dry for several minutes at 37°C and dissolve isolated genomic DNA in 50 µl distilled water. Five microliter genomic DNA was digested by restriction endonuclease *Xho*I (Fermentas Fastdigest, Cat#: FD0694) at 37 °C in a 25 µl system. Agarose gel (1.0-1.2) % was used to do Southern blot with either a ^{32}P dCTP radioisotope labeled telomeric T($G_{1\text{-}3}$) probe (Roche, REF: 11585584001) - or Digoxin (Roche, REF: 11585614910) - labeled telomeric $TG_{1\text{-}3}$ probe.

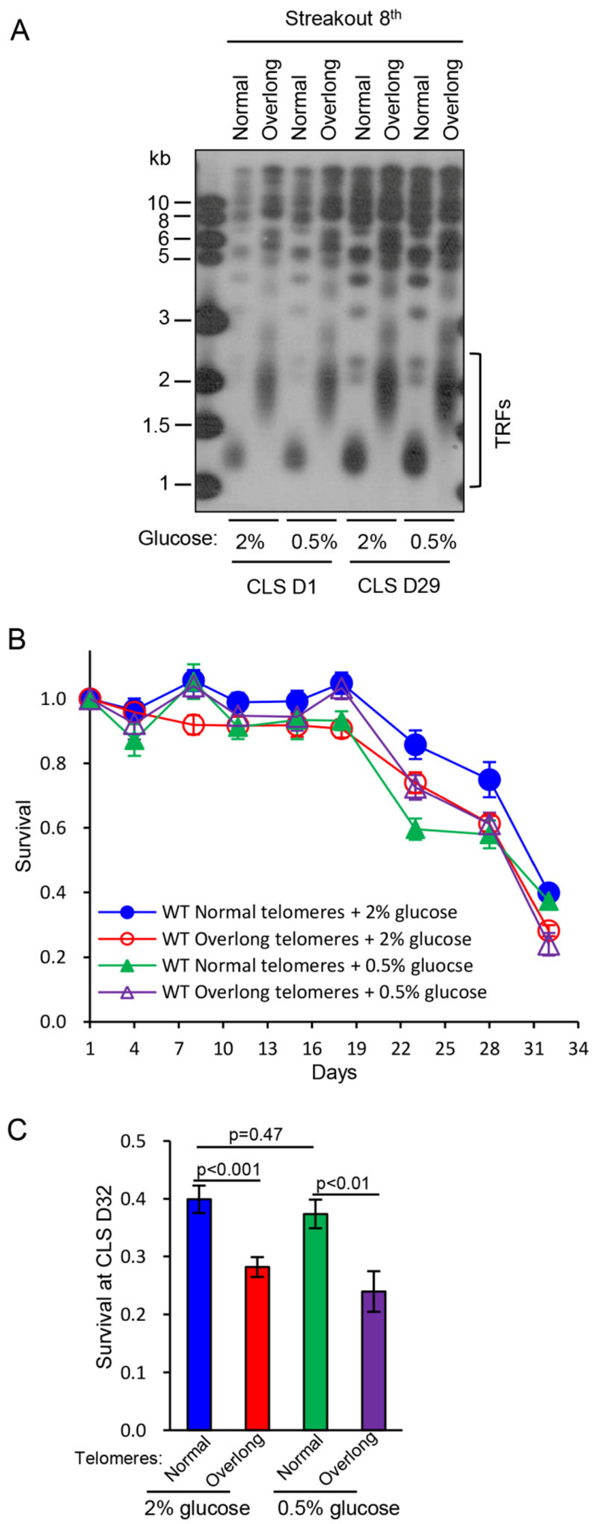

Figure S1. CLS assay of normal- and overlong-telomere cells under moderate calorie restriction condition. (**A**) Telomere length of cells in (**B**) and (**C**) was examined by Southern blot with telomeric TG_{1-3} probe. (**B**) Cells of WT-pRS316 (streakout 8th, normal telomeres) and WT-pRS316-CDC13 -EST2 (streakout 8th, overlong telomeres) were used to perform CLS assay under moderate CR condition in medium buffered to pH 6.0. (**C**) Survival data at CLS D32.

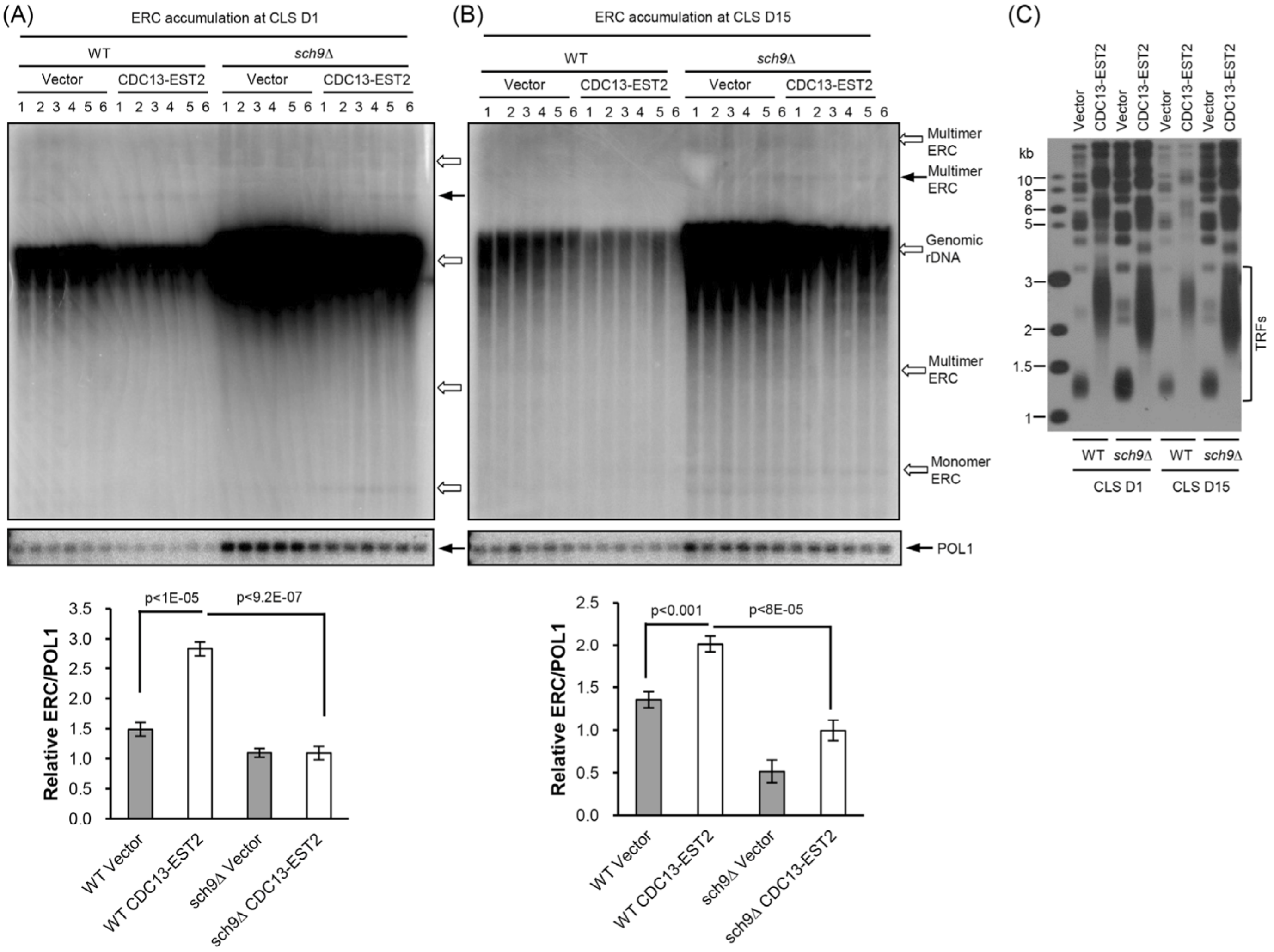

Figure S2. SCH9 deletion suppresses ERCs accumulation. (**A**) and (**B**) Detection of ERC level in young cells of WT-pRS316/ sch9Δ-pRS316 (streakout 14th, normal telomeres) and WT-pRS316-CDC13-EST2/ sch9Δ-pRS316-CDC13-EST2 (streakout 14th, overlong telomeres) at CLS D1 (**A**) and old cells at CLS D15 (**B**) by Southern blot with a probe of 25S sequence (upper panel). One species of multimer ERC (indicated by solid arrow) was quantified (lower panel). POL1 level was used as an internal loading control. Values in the quantification were normalized to POL1 level ± SEM. (**C**) The telomere length of cells used in (**A**) at CLS D1 and (**B**) at CLS D15 was examined by Southern Blot with a telomeric TG1-3 probe.

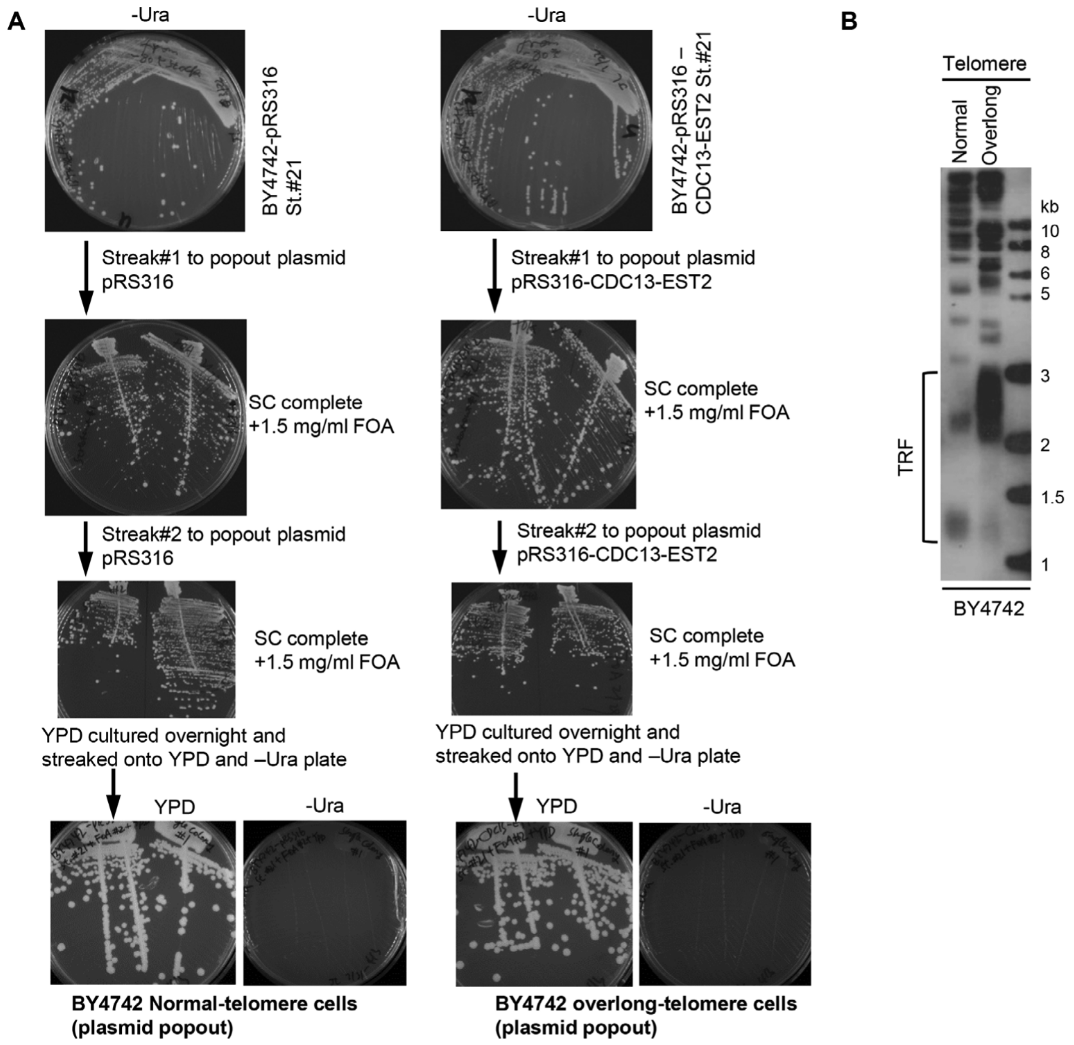

Figure S3. Obtain wild-type BY4742 cells with normal and overlong telomeres but without plasmids carrying either vector or *CDC13-EST2* fusion gene. (**A**) The BY4742-pRS316 (21st streakout) and BY4742-pRS316-CDC13-EST2 (21st streakout) cells stored at -80°C freezer were recovered on fresh –Ura plate and allowed to grow for 3 days. Cells were successively streaked onto FOA plate (1.5 mg/ml) twice every 3 days. Several colonies were suspended into 5ml YPD and incubated overnight and followed by streaking cells onto fresh YPD and –Ura plate. Plates were incubated for 3 days and subjected to photograph. (**B**) Telomere length detection in cells cultured overnight by Southern blot using a telomeric $TG_{1\text{-}3}$ probe.

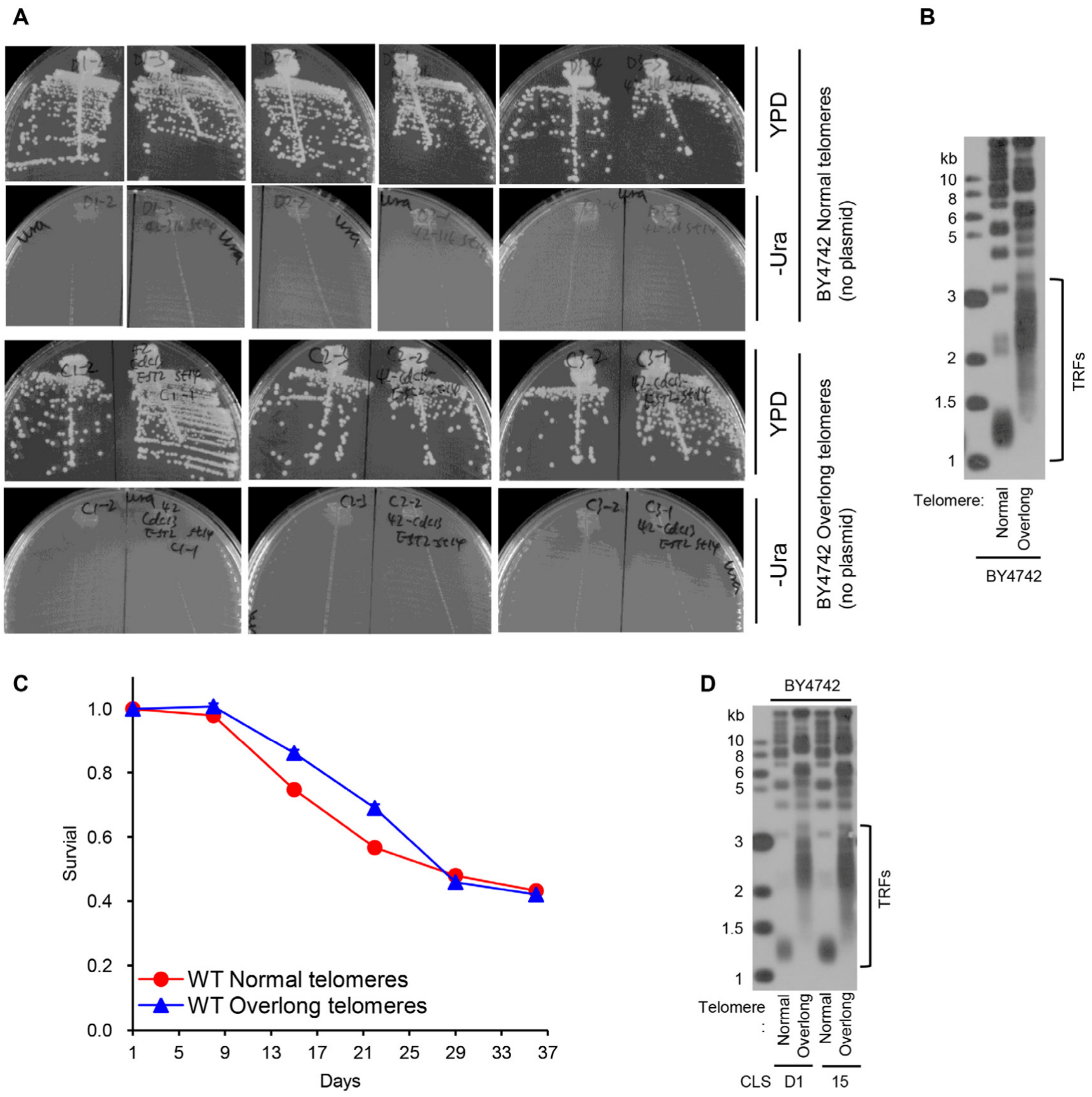

Figure S4. CLS of wild-type BY4742 cells with normal and overlong telomeres but no fusion gene CDC13-EST2. (**A**) BY4742-pRS316 (14th streakout) and BY4742-pRS316-CDC13-EST2 (14th streakout) stored at -80°C freezer were recovered on fresh –Ura plate and allowed to grow for 3 days. Cells were streaked onto to FOA plate (1.5 mg/ml) and allow growing 3-4 days. Several colonies were suspended into 5 ml YPD and incubated overnight and followed by streaking cells onto fresh YPD and –Ura plate. Plates were incubated for 2 days and subjected to photograph. (**B**) Telomere length detection in cells cultured overnight was done by Southern blot with telomeric TG1-3 probe. (**C**) The CLS of wild-type cells (14th streakout) with normal and overlong telomeres. (**D**) Telomere length of cells used in (**C**) at CLS D1 and CLS D15 was examined by Southern Blot with a telomeric TG1-3 probe.

Table S1. Strains and plasmids used in this study.

**Strains**	**Strain Genotype**	**Source**
BY4742	MATα his3Δ1 leu2Δ0 lys2Δ0 ura3Δ0	Euroscarf
hxt13Δ	BY4742 with *hxt13Δ::URA3*	(Chen et al., 2009)
sch9Δ -pRS316	BY4742 with *sch9Δ::HIS3MX6* (pRS316)	This study
sch9Δ-pRS316-CDC13-EST2	BY4742 with *sch9Δ::HIS3MX6* (pRS316-CDC13-EST2)	This study
hxt13Δsch9Δ -pRS316	BY4742 with *hxt13Δ::URA3 sch9Δ::HIS3MX6* (pRS315)	This study
hxt13Δsch9Δ-pRS316-CDC13-EST2	BY4742 with *hxt13Δ::URA3 sch9Δ::HIS3MX6* (pRS315-CDC13-EST2)	This study
fob1Δ	BY4742 with *fob1Δ::KanMX4*	Euroscarf
sir2Δ	BY4742 with *sir2Δ::KanMX4*	Euroscarf
rif1Δ	BY4742 with *rif1Δ::KanMX4*	Euroscarf
BY4742 Streakout#4	BY4742 cells were successively passaged 4 times on YPD plate every 3 days	This study
rif1Δ Streakout#4	BY4742 with *rif1Δ::KanMX4*<br>*rif1Δ* cells were successively passaged 4 YPD plates every 3 days	This study
BY4742 with normal telomeres (streakout#21) (no plasmid)	Transformant BY4742-pRS316 were successively passaged for 21 –Ura plates and stored at -80°C freezer, recovered on –Ura plate and followed by 2 streaks on FOA plate (1.5 mg/ml)	This study
BY4742 with overlong telomeres (streakout#21) (no plasmid)	Transformant BY4742-pRS316-CDC13-EST2 were successively passaged for 21 –Ura plates and stored at -80°C freezer, recovered on –Ura plate and followed by 2 streaks on FOA plate (1.5 mg/ml)	This study
BY4742 with normal telomeres (streakout#14) (no plasmid)	transformant BY4742-pRS316 were successively passaged for 14 –Ura plates, followed by 1 streak on FOA plate (1.5 mg/ml)	This study
BY4742 with overlong telomeres (streakout#14) (no plasmid)	transformant BY4742-pRS316-CDC13-EST2 were successively passaged for 14 –Ura plates, followed by 1 streak on FOA plate (1.5 mg/ml)	This study
**Plasmids**	**Plasmid Description**	**Source**
pRS316	*CEN URA3, empty vector control*	
pRS316-CDC13-EST2	*CEN URA3 CDC13-EST2* fusion expression	(Tong et al., 2011)
pRS315	*CEN LEU2, empty vector control*	
pRS315-CDC13-EST2	*CEN LEU2 CDC13-EST2* fusion expression	This study
pUC19-POL1	For *POL1* as a probe for internal loading control, release by *EcoR*I digestion	(Peng et al., 2015)
pUC19-$TG_{(1-3)}$	*Telomeric probe, release by EcoR*I digestion	(Peng et al., 2015)
pFA6a-HIS3MX6	*HIS3MX6 cassette*	(Longtine et al., 1998)

Table S2. Primers used in this study.

Primer	Sequence
sch9-UP45-HISMX6-F1	GAATTATACTCGTATAAGCAAGAAATAAAGATACG AATATACAATCGGATCCCCGGGTTAATTAA
sch9-DW45-HIS3MX6-R1	AAGAAAAGGAAAAGAAGAGGAAGGGCAAGAGGA GCGATTGAGAAAGAATTCGAGCTCGTTTAAAC
SCH9-5UTR-Fwd	TACTTATTCACATTACGGGTCCAAT
SCH9-3UTR-Rev	ATTCGATGGGATGACAGTTAAGC
HIS3MX6-Fwd	CCAGCCCAAAAAAAGCAAAAAC
HIS3MX6-Rev	AAACACCTTTGGTTGAGGGAAC
SCH9-ORF-Fwd	GAAGTTACAATAATAGAAGCACGTGAC
SCH9-ORF-Rev	CTTTGAGTGTCTTTCTTCTTAACTTGG
HXT13-5UTR-Fwd	TTTCCCCTGGATATATGCGCAA
HXT13-3UTR-Rev	TACGTGTTATGTTCCTTCAATCGC
URA3-ORF-Fwd	ACTACATATAAGGAACGTGCTGCTAC
URA3-ORF-Rev	ATGGCCGCATCTTCTCAAATA
RDN25-Fwd	TGCCCGAGTTGTAATTTGGAGAG
RDN25-Rev	CACAAGGACGCCTTATTCGTATC
FOB1-5UTR-Fwd	TTCATCATACCTAACATTGTGATCG
FOB1-ORF-Fwd	TAAATACGATGGTGTAGTGGCAG
FOB1-ORF-Rev	TAACTATTTAGAGCATACTGAATGCACAG
KanMX4-ORF-Rev	CTGCAGCGAGGAGCCGTAAT
KanMX4-ORF-Fwd	TGATTTTGATGACGAGCGTAAT
SIR2-5UTR-Fwd	CTTTTCCAAGCTACATCTAGCACTC
SIR2-3UTR-Rev	ACCTGCCCTTCTTACATTAAGCTAT
SIR2-ORF-Fwd	CAAGGGGCCAATCATAATCA
SIR2-ORF-Rev	TAACAGTACGGGCATAGTGGA
Rif1-5UTR	TTTTCAGTTCTTTGTGTTTTTCCTC
Rif1-ORF-Fwd	TACACAACGGTAATATTTTCACATCACC
Rif1- ORF-Rev	CACATAAGGAAGCGTCATTTGA

SUPPLEMENTARY REFERENCES

1. Chen X-F, Meng F-L, and Zhou J-Q. Telomere Recombination Accelerates Cellular Aging in *Saccharomyces cerevisiae*. PLoS Genetics. 2009; 5: e1000535.

2. Huang X, Liu J, and Dickson R.C. Down-Regulating Sphingolipid Synthesis Increases Yeast Lifespan. PLoS Genet. 2012; 8:e1002493.

3. Longtine MS, McKenzie Iii A, Demarini DJ, Shah NG, Wach A, Brachat A, Philippsen P, and Pringle JR. . Additional modules for versatile and economical PCR-based gene deletion and modification in Saccharomyces cerevisiae. Yeast. 1998; 14:953-61.

4. Madia F, Gattazzo C, Wei M, Fabrizio P, Burhans WC, Weinberger M, Galbani A, Smith JR, Nguyen C, Huey S, et al. Longevity mutation in SCH9 prevents recombination errors and premature genomic instability in a Werner/ Bloom model system. The Journal of Cell Biology. 2008; 180:67-81.

5. Peng J, He M-H, Duan Y-M, Liu Y-T, and Zhou J-Q. Inhibition of Telomere Recombination by Inactivation of KEOPS Subunit Cgi121 Promotes Cell Longevity. PLoS Genetics 2015; 11:e1005071.

6. Tong X-J, Li Q-J, Duan Y-M, Liu N-N, Zhang M-L, and Zhou J-Q. Est1 Protects Telomeres and Inhibits Subtelomeric Y' Element Recombination. Molecular and Cellular Biology. 2011; 31:1263-74.

7. Wei M, Madia F, and Longo VD. Studying Age-dependent Genomic Instability using the S. cerevisiae Chronological Lifespan Model. J Vis Exp, e3030. 2011;
